# Occlusal Disorders among Patients with Total Clefts of Lip, Alveolar Bone, and Palate

**DOI:** 10.1155/2014/583416

**Published:** 2014-05-27

**Authors:** Anna Paradowska-Stolarz, Beata Kawala

**Affiliations:** ^1^Department of Maxillofacial Orthopedics and Orthodontics, Faculty of Dentistry, Wroclaw Medical University, 50425 Wroclaw, Poland; ^2^Department of Orthodontics, Faculty of Dentistry, Wroclaw Medical University, 26 Krakowska Street, 50425 Wroclaw, Poland

## Abstract

Clefts are common birth defects. They are accompanied by various malformations, including disturbances in facial look as well as skeletal disorders that include malocclusions, most frequently crossbites and class III anomalies. The aim of the study was to present the commonest malocclusions in patients with total cleft of the lip, alveolar bone and palate (*n* = 154) and compare the results to the healthy on-cleft patients (*n* = 151). Normal occlusion, characteristic for I angle class, was observed in 50% of the control group and 30% of the examined. In the examined patients with clefts, most frequently crossbite and open bite on the cleft side was observed. In patients with clefts, only 2 out of 154 patients presented isolated dental anomalies. In healthy individuals the commonest occlusal disorder was distal occlusion and dental anomalies. The commonest malocclusions among patients with clefts are crossbites and class III malocclusions.

## 1. Introduction

Occlusal disorders, that lead to increased orthodontic treatment needs, are a common problem of society. Epidemiologic studies show that in Poland they are observed in 61.8% of youngsters (after [1]). Malocclusions are a common risk factor of functional changes in the stomatognathic system and represent 54% of all causes [2].

Among the patients with clefts, the most common occlusal disorders are crossbites and class III malocclusion. They are classified with IOTN index at stage 5, which represents severe malocclusions that require orthodontic treatment. This concerns 92.4% of men and 71.4% of women. Those characteristics are caused by the three-dimensional maxillary hypoplasia, caused by performed surgical procedures. The dental arch narrowing and shortening is not observed if the cleft was not operated on [3–5]. According to Vettore and Sousa Campos [6] only 25% of cleft patients do not require orthodontic treatment. Among the malocclusions, the most frequent observation is a crossbite on the cleft side and in the incisor region. This is most accented at the canine as the dental arch shape collapses there [6, 7]. The malocclusion is complicated by the asymmetry, especially within the upper dental arch. The asymmetry is the result of the rotation of the parts of clefted maxilla. In one-sided clefts, the bigger part (that includes philtrum and intermaxillary bone) is rotated upward and anteriorly. The smaller part is rotated backwards [8]. According to Lithuanian researchers [9], the severer the cleft is, the more observable the maxillary hypoplasia is.

If as the “occlusal norm” class I according to Angle classification is established, only one-fourth of the cleft patients do not represent malocclusions [6]. According to Wojtaszek-Słomińska [10], crossbites are more often observed in cleft patients, while mesiodistal occlusal anomalies are observed in only one-third of the patients and this number is similar to the one observed in noncleft patients.

When referring to the unilateral cleft patients, GOSLON index to establish occlusal disorders is used. The scale is divided into five categories, representing the severity of malocclusions. GOSLON index 1 means that the patient represents only dental anomalies, while mesiodistal and lateral contacts in occlusion are correct. In stage 2 lateral crossbite with palatotrusion of upper incisors is observed. GOSLON index 3 means that there is a lateral crossbite that reaches at least to tette-a-tette occlusion of the incisors or complete crossbite of one side is observed. GOSLON 4 and GOSLON 5 refer to severe malocclusions with crossbites that include the bone basis and require orthognathic treatment. In GOSLON 5 also an open bite is observed. For bilateral clefts a similar index is used and to facilitate the classification it is also called GOSLON, though real GOSLON index was created for unilateral cleft patients only. In GOSLON 1 normal occlusion with dental anomalies is observed (maximum one tooth could be in a crossbite). In GOSLON 2, pseudodistoclussion or deep bite is observed. GOSLON 3 stands for partial crossbite in either frontal or lateral part (unilateral or bilateral). As in unilateral clefts, GOSLON 4 and GOSLON 5 represent severer malocclusions with pseudomesioclussion and open bite that require orthognathic treatment. It is estimated that GOSLON index of value 3 or higher is observed in 60–70% of the cleft patients [10–13]. One should remember that even though orthodontic diagnosis is based on the relations of the jaws, the treatment planning should include the natural head position and the soft tissues as a part of stomatognathic balance [14, 15].

The aim of the study was to estimate the occlusal problems in patients with total clefts of the lip, alveolar bone, and palate and compare them to healthy individuals.

## 2. Material and Methods

Based on the dental casts and panoramic and cephalometric radiographs, the orthodontic diagnosis for 154 patients with unilateral and bilateral total clefts of lip and palate was made. The cleft was isolated (no other birth defect was present in the patients). Bilateral cleft (BCLP) was observed in 36 patients (17 women and 19 men). Unilateral clefts were observed on the left side (CLP-L) in 87 patients (36 women and 51 men) and on the right side (CLP-R) in 31 cases (8 women and 23 men). The control group comprised of 151 patients (96 women and 55 men) with orthodontic treatment needs and without any developmental anomalies. None of the patients had undergone orthodontic treatment with any type of fixed appliance.

The plaster casts were used to establish Angle and canine class. Supplementing the diagnosis with X-rays allowed for stating the orthodontic diagnosis. The diagnosis was made according to three planes: anteroposterior, vertical, and horizontal. Frequency tables were used to reveal the differences between the examined groups.

## 3. Results

In the examined group, all of the patients presented fully erupted permanent dentition, which is understood by presence of teeth to first molars at least (in ca. 5% of the examined patients with cleft impations of the permanent teeth were present). Congenital lack of tooth buds was observed in 20% of patients with BCLP and 37.84% of male and 47.73% female patients with unilateral clefts. Hypodontia in patients without congenital deformities was observed twenty times rarer in boys and ten times rarer in girls. Hypodontia in most cases referred to upper lateral incisor on the cleft side. Hyperdontia was observed in 15% of patients with unilateral clefts and 20% of patients with bilateral type of deformity and referred to the lateral incisor of the cleft side.

In all of the examined groups Angle and canine class I are the most common observation. The normal occlusion is observed in 50% of healthy patients, while it is observed in ca. 30% of cleft patients. Among the cleft patients the most common observations are class III malocclusions, while it is observed in healthy individuals only in 10% of cases (Tables 1 and 2).

Tables 3 and 4 represent observations on malocclusions in patients with clefts, according to Orlik-Grzybowska.  Table 3 concerns a group of boys, Table 4—girls.

As presented in Table 3, in a control group of boys the most frequent malocclusions are class II (distal occlusions) that are observed in nearly 42% of the examined boys. In patients with clefts those are observed in less than 10%. Class III malocclusions are observed in patients with unilateral types of clefts more frequently than in healthy individuals (more than 30% in CLP-R and 20% in CLP-L versus less than 10% in a control group of boys). In patients with clefts, transverse malocclusions are the most common problem—the most common observations are partial crossbites that refer to the cleft side. Isolated dental anomalies with normal occlusion are observed in 35% of healthy patients, while they are not observed in cleft patients. Deep bites are a common observation in BCLP and they are observed in more than 25% of the cases.

Table 4 presents the types of malocclusion in a group of girls. In a group of girls without any birth defects, as in a group of boys, the most common observation is distal malocclusions (observed in almost 50% of girls). Class III malocclusions in this group are observed only in ca. 5%, while they are observed in 25% of CLP-L and nearly 10% of BCLP. As observed in boys, also in girls the most commonly observed malocclusions in cleft individuals are transverse ones, almost only on the affected side. Also a common observation in a group of patients with clefts is open bite (13–25%). Isolated dental anomalies are observed in 33% of healthy girls and barely observed in cleft individuals.

## 4. Discussion

As in other researches, the cleft was more frequently observed on the left side than in the right side, which is the result of embryologic fusion of the palate and the fact that in case of the right side this process lasts longer and even if the disturbing factor occurs at the pregnant woman, the fusion of the lip on the right side might occur later in the development [16–20].

From the research, patients with clefts present different types of malocclusion than patients without this birth defect. A study conducted in Hong Kong [4] showed that the majority of patients with clefts were characterized by severe malocclusions in the early phases of development of occlusion (mixed dentition)—the bite defects were observed in 92.3% of men and 71.4% of women. Among the malocclusions, the most common mentioned one was the mesial molar relationship (69.2% men and 57.1 women), lateral crossbite, diastema, and medial line disturbances. Brazilian studies [6] indicate that only every fourth child with cleft does not have malocclusion. In our study, only 2 of 154 patients with cleft defect were free of severe malocclusions and presented only isolated dental anomalies. Class III malocclusions, according to Swanson et al. [16], are unilateral in approximately one-third of the patients with clefts and are the result of growth inhibition and rotation of the clefted maxilla. Additionally, the deterioration of malocclusion occurs during the pubertal growth spurt (ca. 8–10 years of age), when the underdevelopment of maxilla is accentuated [17, 18]. The caries decay and premature loss of deciduous teeth that is more frequently observed in cleft patients, leads to migration of teeth, and worsens occlusion [21]. In our study mesial occlusion was observed in 19.89% of patients with clefts compared to 6.62% in patients with no birth defects.

Class II malocclusions predominate among the control group and occur in nearly half of the girls and 40% of the boys. Data found in the current literature suggest lower incidence of class II malocclusions, ranging around 10–20% [22–25]. A similar result of more than fifty percent turnout prevalence of class II was observed in Finnish adolescents, but these studies were performed in children with mixed dentition [26].

The skeletal relation of jaws determines occlusal relations, which is why it is so important when orthodontic treatment is planned. Only a proper occlusion may lead to balance within the orthognathic system, but nowadays orthodontic treatment planning also takes into account also soft tissues and their esthetics. The soft tissues and profile might be estimated on photographs, but also a cephalometric X-ray is enough to assess patient's profile and soft tissues appearance [14, 15]. Moreover, improper occlusion may lead to many problems, including severe temporomandibular disorders. Deep bites and crossbites are the most common malocclusions in patients suffering from the temporomandibular joint disorders that may require treatment [27, 28]. Our observations may lead to the conclusions that patients with clefts are a predominant group to temporomandibular disorders, as they present most frequently crossbites (additionally, in patients with BCLP deep bites are observed more frequently).

Our observations of bite symmetry show that patients with clefts represent asymmetrical types of bite—usually crossbite or open bite is more frequently observed on the cleft side. The observations of other authors [29] concerning asymmetry in patients with clefts lead to the conclusion that temporomandibular fossa lies lower at the cleft side and is steeper there.

## 5. Conclusions

Patients with clefts more frequently represent crossbites than the general population, while isolated dental anomalies are not a characteristic of these patients. In noncleft patients distal occlusion is a common observation, while in patients with clefts class III malocclusions dominate.

## Figures and Tables

**Table 1:** Angle and canine classification: right side.

	I Angle class *n* (%)	I canine class *n* (%)	II Angle class *n* (%)	II canine class *n* (%)	III Angle class *n* (%)	III canine class *n* (%)	Number of the examined cases [*n*]
Men							
CLP-R	10 (43.48)	11 (47.83)	5 (21.74)	5 (21.74)	7 (30.43)	6 (26.09)	23
CLP-L	17 (33.33)	23 (45.1)	14 (27.45)	11 (21.57)	20 (39.21)	16 (31.37)	51
BCLP	7 (36.84)	5 (26.32)	7 (36.84)	10 (52.63)	5 (26.32)	4 (21.05)	19
controls	31 (56.36)	32 (58.18)	19 (35.55)	19 (35.55)	5 (9.09)	4 (7.27)	55

Women							
CLP-R	4 (50.00)	4 (50.00)	1 (12.50)	2 (25.00)	3 (37.50)	2 (25.00)	8
CLP-L	16 (44.44)	15 (41.67)	9 (25.00)	11 (30.56)	8 (22.22)	7 (19.44)	36
BCLP	9 (52.94)	5 (29.41)	6 (35.29)	6 (35.29)	3 (17.65)	4 (23.53)	17
controls	52 (54.17)	47 (48.96)	36 (37.50)	43 (44.79)	9 (9.38)	4 (4.17)	96

**Table 2:** Angle and canine classification: left side.

	I Angle class *n* (%)	I canine class *n* (%)	II Angle class *n* (%)	II canine class *n* (%)	III Angle class *n* (%)	III canine class *n* (%)	Number of the examined cases [*n*]
Men							
CLP-R	7 (30.43)	7 (30.43)	5 (21.74)	5 (21.74)	10 (43.48)	10 (43.48)	23
CLP-L	19 (37.25)	9 (17.65)	16 (31.37)	19 (37.25)	17 (33.33)	17 (33.33)	51
BCLP	5 (26.32)	4 (21.05)	9 (47.37)	11 (57.89)	5 (26.32)	4 (21.05)	19
controls	28 (50.91)	28 (50.91)	21 (38.18)	20 (36.36)	6 (10.91)	6 (10.91)	55

Women							
CLP-R	5 (62.50)	4 (50.00)	2 (25.00)	2 (25.00)	1 (12.50)	2 (25.00)	8
CLP-L	15 (41.67)	11 (30.56)	8 (22.22)	13 (36.11)	10 (27.78)	9 (25.00)	36
BCLP	8 (47.06)	7 (41.18)	7 (41.18)	8 (47.06)	2 (11.76)	2 (11.76)	17
controls	53 (55.21)	49 (51.04)	37 (38.54)	44 (45.83)	6 (6.25)	2 (2.08)	96

**Table 3:** Malocclusions in a group of boys.

Diagnosis	CLP-R		CLP-L		BCLP		Controls	
	*n* = 23	%	*n* = 51	%	*n* = 23	%	*n* = 51	%
Class II malocclusions								
Distoclusion with protrusion of upper incisors	1	4.35	5	9.80	1	5.26	20	36.36
Distoclusion with retrusion of upper incisors	0	0	1	1.96	1	5.26	1	1.82
Partial distoclusion	0	0	0	0	0	0	2	3.67
Pseudodistocclusion	0	0	0	0	0	0	0	0
Retrogenia	1	4.35	0	0	0	0	0	0
Class III malocclusions								
Total mesiocclusion	1	4.35	0	0	0	0	4	7.27
Partial mesiocclusion	0	0	1	1.96	0	0	1	1.82
Pseudomesiocclusion	6	26.09	10	19.61	1	5.26	0	0
Progenia	0	0	0	0	0	0	2	3.67
Vertical malocclusions								
Open bite: partial, anterior	0	0	2	3.92	0	0	3	5.45
Total open bite	0	0	0	0	0	0	1	1.82
Open bite: lateral, right-sided	0	0	1	1.96	0	0	1	1.82
Open bite: lateral, left-sided	1	4.35	4	7.84	2	10.53	0	0
Deep bite	0	0	0	0	5	26.32	4	7.27
Transverse malocclusions								
Crossbite: partial, anterior	5	21.74	8	15.69	0	0	2	3.67
Total crossbite	5	21.74	7	13.73	4	21.05	0	0
Crossbite: partial, right-sided	17	73.91	8	15.69	7	36.84	5	9.09
Crossbite: partial, left-sided	9	39.13	31	60.79	10	52.63	6	10.91
tette-a-tette	3	13.04	5	9.80	0	0	0	0
Lingual occlusion	0	0	1	1.96	0	0	0	0
Class I malocclusions								
Isolated teeth anomalies	0	0	0	0	0	0	19	34.55

**Table 4:** Malocclusions in a group of girls.

Diagnosis	CLP-R		CLP-L		BCLP		Controls	
	*n* = 8	%	*n* = 36	%	*n* = 17	%	*n* = 96	%
Class II malocclusions								
Distoclusion with protrusion of upper incisors	0	0	4	11.11	1	5.88	34	35.42
Distoclusion with retrusion of upper incisors	0	0	0	0	4	23.53	8	8.33
Partial distoclusion	0	0	0	0	0	0	4	4.17
Pseudodistocclusion	0	0	0	0	0	0	1	1.04
Retrogenia	0	0	0	0	0	0	0	0
Class III malocclusions								
Total mesiocclusion	0	0	1	2.78	0	0	1	1.04
Partial mesiocclusion	0	0	1	2.78	1	5.88	0	0
Pseudomesiocclusion	0	0	7	19.44	7	41.18	1	1.04
Progenia	0	0	0	0	0	0	2	2.08
Vertical malocclusions								
Open bite: partial, anterior	0	0	1	2.78	1	5.88	6	6.25
Total open bite	0	0	0	0	0	0	3	3.13
Open bite: lateral, right-sided	2	25.0	0	0	2	11.76	0	0
Open bite: lateral, left-sided	0	0	5	13.89	5	29.41	2	2.08
Deep bite	0	0	0	0	0	0	1	1.04
Transverse malocclusions								
Crossbite: partial, anterior	2	25.0	3	8.33	5	29.41	5	5.21
Total cross bite	1	12.5	9	25	10	58.82	0	0
Crossbite: partial, right-sided	6	75.0	7	19.44	8	47.05	8	8.33
Crossbite: partial, left-sided	3	37.5	21	58.33	14	82.35	3	3.13
tette-a-tette	0	0	5	13.89	5	29.41	2	2.08
Lingual occlusion	0	0	0	0	1	5.88	32	33.33
Class I malocclusions								
Isolated teeth anomalies	0	0	1	2.78	0	0	6	6.25
